# Psychosis-Related Refusal of Life-Sustaining Insulin: Clinical and Legal Challenges at the Mental Health Act–Mental Capacity Act Interface

**DOI:** 10.1192/bjo.2026.11779

**Published:** 2026-06-30

**Authors:** Mariana Galvao de Oliveira, Melisa Kanber

**Affiliations:** South London and Maudsley NHS Foundation Trust, London, United Kingdom

## Abstract

**Aims:**

**Background**

People with severe mental illness experience significantly higher rates of physical morbidity and premature mortality, with diabetes mellitus being a major contributor. When psychotic symptoms impair insight and decision-making, refusal of essential medical treatment can present acute and recurrent risks. In such situations, clinicians must navigate the interface between the Mental Health Act (MHA) and the Mental Capacity Act (MCA), two statutory frameworks that were not designed to address sustained community-based enforcement of physical healthcare in the context of fluctuating mental capacity.

**Methods:**

**Case Report**

We describe the case of a patient with a chronic psychotic disorder and insulin-dependent diabetes mellitus who repeatedly refused insulin during psychotic relapses, leading to recurrent episodes of diabetic ketoacidosis requiring emergency hospital admission. During periods of florid psychosis, the patient lacked capacity to make decisions regarding insulin administration, with refusal directly driven by delusional beliefs. Despite multidisciplinary involvement, optimisation of psychiatric treatment, and repeated capacity assessments, adherence could not be sustained in the community. Each crisis was managed reactively through emergency admission, with no clear mechanism for anticipatory or preventative intervention once the immediate medical emergency resolved.

**Results:**

**Discussion**

This case highlights a recurrent clinical and ethical dilemma at the MHA–MCA interface. While the MCA permits best-interests’ decisions during acute incapacity, it offers limited scope for proactive intervention when capacity fluctuates, and risk is foreseeable but not immediate. Conversely, the MHA allows compulsory treatment for mental disorder but does not clearly support sustained enforcement of treatment for physical illness, even when refusal is a direct consequence of psychosis. This case does not advocate for routine compulsory treatment of physical illness under the MHA. Rather, it illustrates a structural gap in existing legal frameworks when psychosis-driven refusal leads to repeated, predictable medical crises that are addressed only reactively.

**Conclusion:**

Psychosis-related refusal of life-sustaining medical treatment exposes limitations in current statutory frameworks, particularly for individuals with recurrent incapacity and high physical health risk. Earlier, legally supported escalation: including proactive capacity planning, structured review of statutory options, and consideration of Court of Protection involvement may help reduce preventable harm and improve parity between mental and physical healthcare. This case underscores the need for clearer guidance and integrated approaches to managing sustained physical health risk in the context of severe mental illness.

